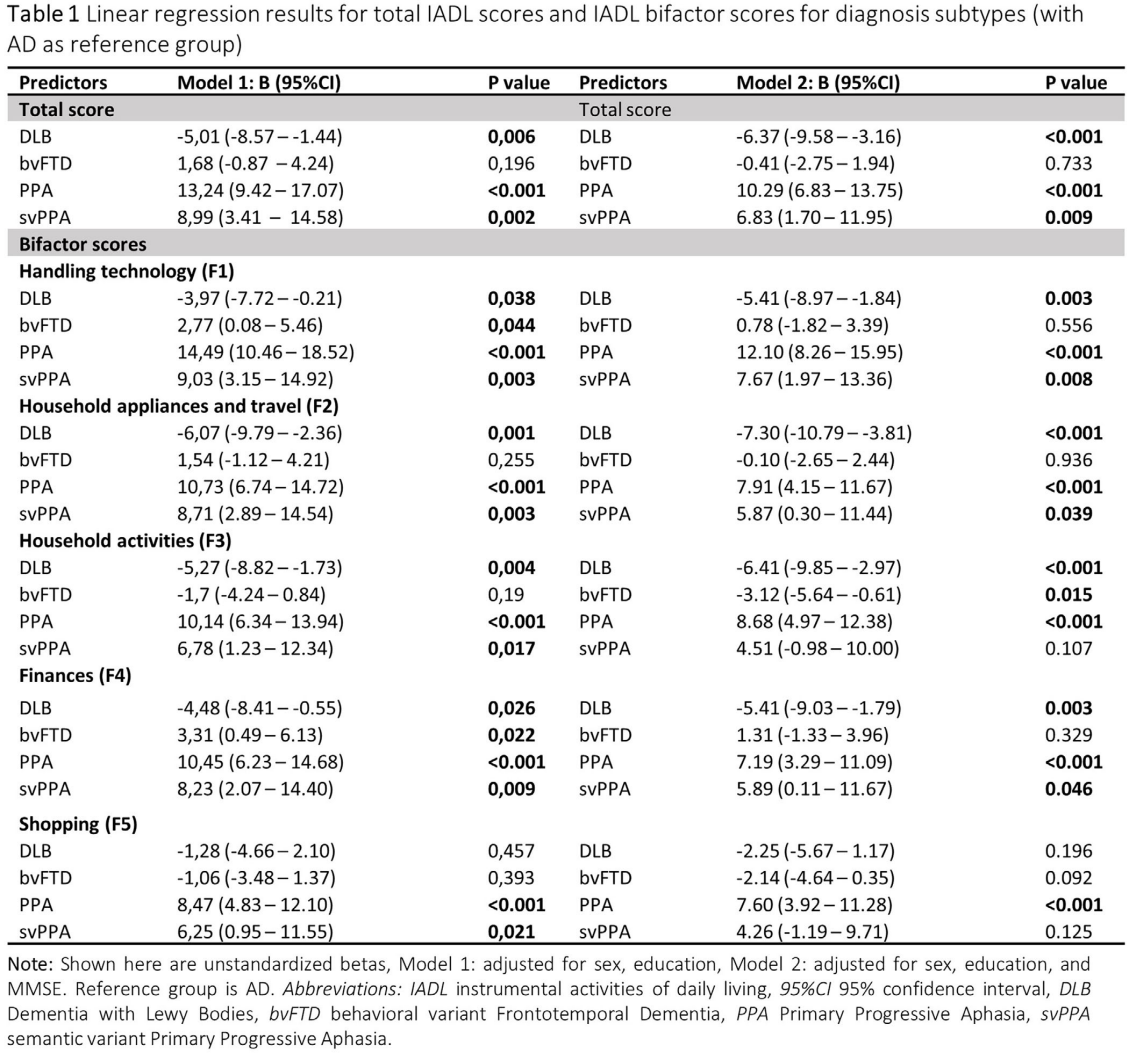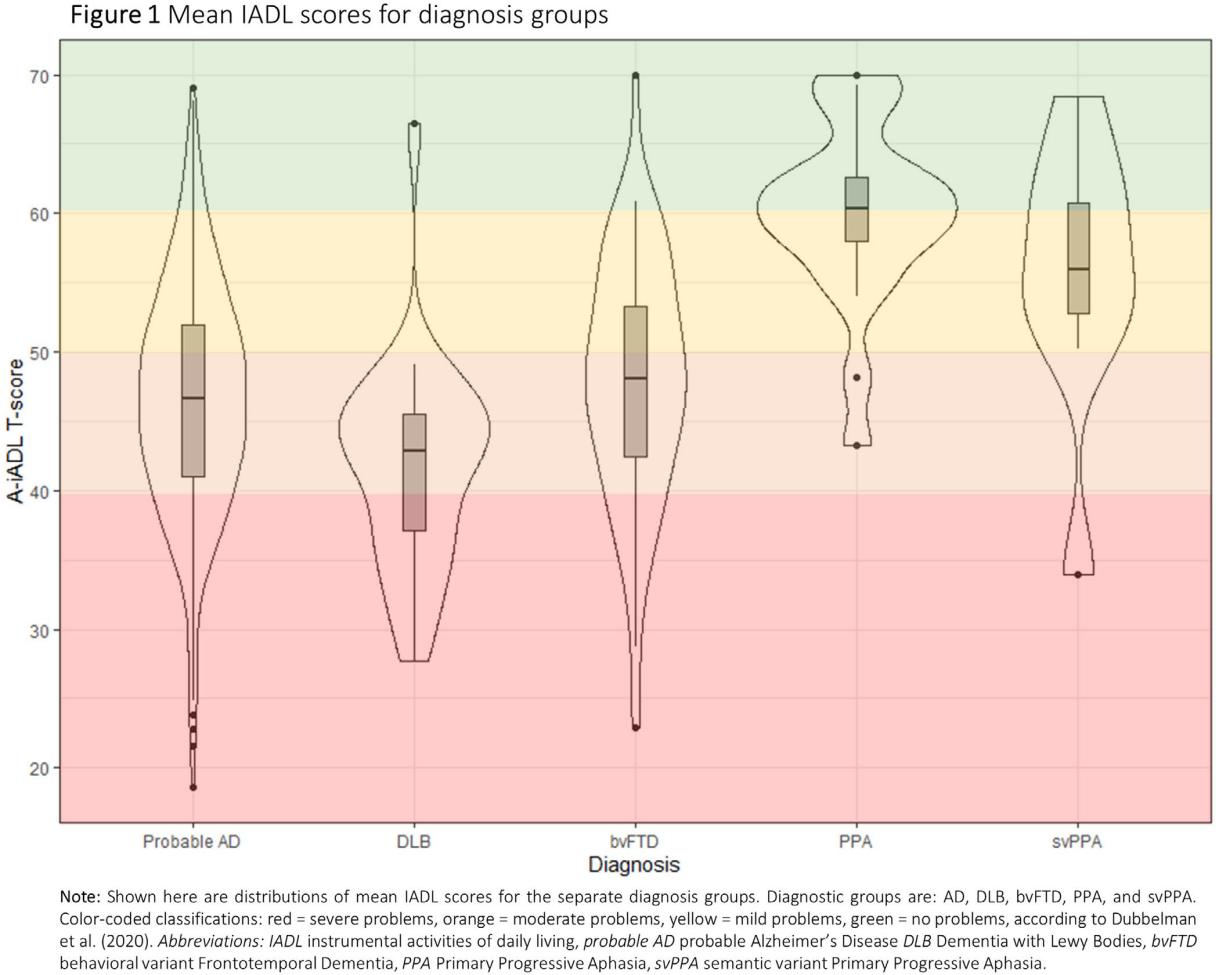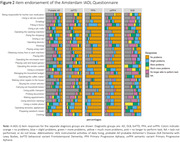# Everyday functioning in young onset dementia: differences in diagnostic groups independent of disease stage

**DOI:** 10.1002/alz.092202

**Published:** 2025-01-03

**Authors:** Emma Weltings, Merel C. Postema, Mukrabe E. Tewolde, Flora H. Duits, Afina Willemina Lemstra, Wiesje M. van der Flier, Yolande A.L. Pijnenburg, Sietske A.M Sikkes

**Affiliations:** ^1^ Alzheimer Center Amsterdam, Neurology, Vrije Universiteit Amsterdam, Amsterdam UMC, Amsterdam Netherlands; ^2^ Amsterdam Neuroscience, Neurodegeneration, Amsterdam Netherlands; ^3^ Alzheimer Center Amsterdam, Neurology, Vrije Universiteit Amsterdam, Amsterdam UMC location VUmc, Amsterdam Netherlands; ^4^ Department of Epidemiology and Data Science, Vrije Universiteit Amsterdam, Amsterdam UMC, Amsterdam Netherlands; ^5^ Amsterdam Neuroscience, Neurodegeneration, Amsterdam, Noord‐Holland Netherlands; ^6^ Faculty of Behavioural and Movement Sciences, Vrije Universiteit Amsterdam, Amsterdam Netherlands

## Abstract

**Background:**

Young onset dementia (YOD) is characterized by an atypical clinical manifestation, and it is unclear to what extent impairments in everyday functioning are part of this manifestation. This study aims to describe the prevalence and differences of difficulties in instrumental activities of daily living (IADL) in YOD.

**Methods:**

In this cross‐sectional study, 394 subjects with sporadic YOD (onset<65 years,*mean(M)age 58.4±standard deviation(SD)4.4 years;49.2% female*) were included from the Amsterdam Dementia Cohort (ADC). Diagnoses were established in multidisciplinary consensus meetings according to disease‐specific diagnostic criteria. Everyday functioning was assessed using the proxy‐based Amsterdam IADL Questionnaire (A‐IADL‐Q). Linear regression analyses were performed to assess differences between YOD subtypes in IADL with group (YOD subtypes with AD as reference group) and IADL as outcome, adjusted for age, sex, education and disease severity (Mini Mental State Examination). A bifactor analysis was performed to identify item clusters and explore differences between groups (using linear regression).

**Results:**

The majority of sporadic YOD were diagnosed with Alzheimer’s Disease (AD,*n* = 291(74%)), followed by Dementia with Lewy Bodies (DLB,*n* = 25(6%)), behavioral variant Frontotemporal Dementia (bvFTD,*n* = 49(12%)), Primary Progressive Aphasia (PPA,*n* = 20(5%)) and semantic variant Primary Progressive Aphasia (svPPA,*n* = 9(2%)). Most IADL difficulties were observed in DLB ((M) A‐IADL‐Q 41.8±(SD)7.78, reflecting moderate problems), and the fewest in PPA ((M)59.9±(SD)6.62, reflecting no problems*)*(Figure 1). When compared to AD (reference group), multiple linear regression analysis confirmed lower IADL scores for DLB (β = ‐6.37, SE = 1.63,*p*≤0.001), and higher for PPA (β = 10.29, SE = 1.76, p≤0.001), and svPPA (β = 6.83, SE = 2.61,*p* = 0.009), after adjustment for all covariates and confirmed in post‐hoc testing (Table 1). The bifactor analysis showed good reliability of the A‐IADL‐Q (Omega (ω_total) = 0.833), suggesting the presence of an underlying unimodal construct. Additional item clusters (Table 1) showed differences across groups, e.g. bvFTD showed more impairments in household activities compared to AD (β[95%CI] = ‐3.12 [‐5.64 – ‐0.61]). Further activity‐level differences can be found in Figure 2.

**Conclusions:**

Our results suggest that YOD‐subtypes differ in everyday functioning difficulties, independent of disease stage. These findings contribute to the understanding of everyday functioning difficulties in YOD, ultimately contributing to earlier recognition of the disease and personalized care.